# Sharing own story telling during COVID-19’s italian lockdown: An experience with schizophrenic outpatients

**DOI:** 10.1192/j.eurpsy.2021.796

**Published:** 2021-08-13

**Authors:** A. Gentile, I. Matarazzo, S. Marini

**Affiliations:** Centro Di Salute Mentale, AsreM, Termoli, Italy

**Keywords:** COVID-19, Narrative Psychiatry

## Abstract

**Introduction:**

Affective flattening is one of the main symptoms in Schizophrenia, several studies highlighted the importance of social skills training in improving negative symptoms. However, Covid-19 pandemic is changing our life with limitations in social contacts and in psychosocial rehabilitation; pre COVID-19 strategies should be implemented with new ones.

**Objectives:**

To evaluate the practicability of a narrative method in improving affective flattening, general social skills in stable outpatients with a diagnosis for schizophrenia during Italian lockdown in March – April 2020

**Methods:**

Outpatients with a stable psychopathology have been involved in a narrative project during lockdown. We asked to patients to write daily a story telling about their experience and emotions and send us their diary. Every week the diary has been used to discuss their story telling in group in a web conference. At end of the experience we administered a survey about the enjoyment and the subjective benefits.

**Results:**

From ten people with a known psychopathology we recruited six patients. All participants completed the project and all of them referred for a subjective benefit as to feel more reassured by the contact with their psychiatrist. Four patients explicated initial discomfort about share their experiences in group. One patient started to share his own thoughts about mental disease on social media.

**Conclusions:**

Narrative Psychiatry might be a pragmatic opportunity to implement conventional strategies to contrast affective flattening and negative symptoms in Schizophrenia. Sharing digital story telling is a useful method in lockdown and general social restriction condition.

